# Antioxidant Capacity and Phytonutrient Content in the Seed Coat and Cotyledon of Common Beans (*Phaseolus vulgaris* L.) from Various Regions in Mexico

**DOI:** 10.3390/antiox8010005

**Published:** 2018-12-25

**Authors:** Celia Chávez-Mendoza, Karla Ivonne Hernández-Figueroa, Esteban Sánchez

**Affiliations:** Centro de Investigación en Alimentación y Desarrollo A.C., Delicias, Chihuahua 33089, Mexico; celia.chavez@ciad.mx (C.C.-M.); a281607@uach.mx (K.I.H.-F.)

**Keywords:** common bean, Mexico, minerals, seed coat, cotyledon, nutraceuticals

## Abstract

The common bean is a good source of protein and bioactive substances giving it a large antioxidant capacity. The extensive variability of bean genotypes creates the need to characterize them with regard to their nutritional value as a tool in biofortification programs. The purpose of this study was to obtain the antioxidant capacity and phytonutrient content both in the seed coat and the cotyledon of 12 common bean varieties from different regions in Mexico. In the case of the whole seed, lightness (L*), a* (red-purple) and b* (yellow-purple) color coordinates were determined, as well as the chroma and hue angle. In the case of the seed coat and the cotyledon, the protein content, the phytonutrient content and the antioxidant capacity (2,2-diphenyl-1-picrylhydrazyl (DPPH)) were evaluated. A significant difference was observed (*p* ≤ 0.05) among bean varieties and between seed coat and cotyledon in all variables evaluated. Cotyledon showed a higher content of protein, H, Ni, Zn, Cu, N, P, K S and Mn, while the seed coat showed a higher content of Fe, Ca and Mg and a greater antioxidant capacity (59.99%). The Higuera Azufrado bean variety stood out as having a higher content of N, S and protein. We have concluded that the nutritional characterization performed on Mexican bean varieties represents a valuable tool for genetic enhancement programs and crop biofortification strategies.

## 1. Introduction

The common bean is a legume pertaining to the Phaseoleae family and to the Papilionoideae sub-family. The common bean originated in Mesoamerica and was later domesticated between 5000 and 2000 B.C. at two locations in the Americas: Mesoamerica (Mexico and Central America) and the Andes (South America). Starting with wild beans, two different domesticated gene pools thrived, namely, the Mesoamerican and the Andine varieties [1]. Legumes represent the first consumed vegetable group in human nutrition, especially among the low-income population in developing countries. Legumes are a good source of protein, providing more than 2 to 3 times the protein content found in cereals. Also, legumes are rich in dietary fiber and starch [2]. Moreover, the common bean contains a large number of bioactive substances, including enzyme inhibitors, lectins, phytates, oligosaccharides and phenolic compounds [3]. Phenolic compounds are mainly compounds in legumes responsible for the antioxidant potential attributed to these seeds, which possess a wide array of pharmacological and medicinal properties, such as anti-inflammatory, anti-carcinogenic and vasodilation activities [4]. In addition, the common bean possesses an extensive variety of flavonoids, such as anthocyanins, proanthocyanidins, flavonols, phenolic acids and isoflavones. Thus, common bean consumption is associated with various physiological and health-related benefits, such as preventing cardiovascular disease, obesity, diabetes mellitus and cancer [5]. It is also known to contain unsaturated fatty acids (linoleic acid), vitamins and minerals [6]. The common bean is estimated to contain 4 to 10 times more iron and 2 to 3 times more Zn than rice [7]. Moreover, bean consumption in Latin America provides 8–27% of potassium needed to satisfy daily nutrition requirements. This legume contains up to three times more potassium than bananas [8]. It also has a high phosphorus content in which a high genetic variation has been observed. Thus, it has been reported that common bean seeds pertaining to the Mesoamerican gene pool normally show a higher phosphorus content than the seeds from Andine gene pools [9]. 

Furthermore, the common bean grain is formed by the seed coat (which represents approximately 9% of the total dry matter contained in the seed), the cotyledon (which represents approximately 90% of total dry matter), and the embryonic axis, (which only represents 1% of total dry matter). More than 80% of calcium and only 1.9–3.6% of phosphorus is found in the seed coat [9]. The bean color has also been directly related with bioactive compounds, such as condensed tannins, which are found in higher concentrations in beans having a pink seed coat as compared to those having a yellow seed coat [10]. Phenolic compounds and flavonoids are found in higher quantities in beans having dark seed coats as compared to pale bean varieties, providing a huge antioxidant capacity [11,12]. These compounds as well as anthocyanins result in red, black or pink colorations in bean varieties [13]. Anthocyanins are usually found in the seed coat, although they have also been found in the hypocotyl and the cotyledon [14]. 

Moreover, the genotype and the environment affect the accumulation of minerals in common beans [15]. This grain has a large variety of phenotypes in terms of seed, color and mineral composition, which reveals a rich genetic diversity among species [16]. Mexico has been recognized as the central origin of the common bean because 47 of the 52 varieties classified under the *Phaseolus* genus were identified in Mexico [17]. However, bean consumption habits vary from one region to another and are influenced by the economic situation, local traditions [10], and the color of bean grains [18]. Mexico is home to a widely diverse variety of beans. Light yellow beans (Canary, Azufrado, Mayocoba) predominate in the Northwestern region, while pinto, Bayo, Flor de Mayo, garbancillo, and black beans predominate in the North-Central region. On the other hand, several local bean varieties such as Flor de Mayo, Flor de Junio, black beans and Criollas varieties (such as Rebosero, Garrapato, Coconita, etc.) prevail in the Central region. In the humid tropical region, small grain beans such as black beans and opaque beans are more common. This diversity results from the fact that this species can adapt to tropical and mild climates in various altitudes. Moreover, beans grow in almost all types of soil [19]. The importance of common bean in the diet of Mexico and other countries, in addition to the extensive diversity of existing varieties and its high content of nutraceutical compounds raised the need to conduct a research work to characterize the bean varieties produced and largely consumed in Mexico. Therefore, the purpose of this study is to characterize the phytonutrient content and antioxidant capacity contained in the seed coat and cotyledon of twelve common bean varieties grown in different regions throughout Mexico and one variety grown in Cuba.

## 2. Results and Discussion

### 2.1. Measuring the Antioxidant Capacity (DPPH) and Total Phenols of Common Bean Varieties

Table 1 shows the antioxidant capacity and total phenols observed in the seed coat and cotyledon of common bean varieties. The statistical analysis showed a significant difference (*p* ≤ 0.05) both between seed fractions and between bean varieties for both variables. 

The antioxidant capacity seen in the common bean seed coat evaluated ranged from 23.86% to 84.10%. The antioxidant capacity seen in the cotyledon ranged from 0.66% to 29.77%.

The seed coat showed a greater antioxidant capacity than the cotyledon in all bean varieties evaluated. On average, the percent inhibition observed in the seed coat was 59.99%, while the percent inhibition observed in cotyledon was 7.40%. Similar behavior was observed in Brazilian common bean varieties by Gálvez et al. [12], who found a higher content of flavonoids and phenolic compounds in this part of the seed as compared to the cotyledon, thus giving it a greater antioxidant capacity. Moreover, these authors found a greater number of anthocyanins in black beans and red beans, as well as a greater number of kaempferol glycosides in yellow beans, thus reaching the conclusion that these compounds confer these shades to bean seeds.

The Ojo de Cabra bean variety grown in San Francisco de Conchos, Chihuahua (spotted), the Negro 8025 variety (Durango, Instituto Nacional de Investigaciones Forestales, Agricolas y Pecuarias, INIFAP), black), the Flor de Mayo variety Durango INIFAP, spotted) and the Negro San Luis bean variety (Durango INIFAP, black) showed the greatest antioxidant capacity in their seed coat as compared to all varieties evaluated, while the Higuera Azufrado variety (from Guasave, Sinaloa, Mexico, yellow), the Cuba-V7 variety (from San José Las Lajas, Cuba, black), and the Alubia bean variety (from Guadalajara, Jalisco, Mexico, white) showed the lowest values, having no statistical difference. Likewise, the Pinto Saltillo variety (from Vergelitos Sombrerete, Zacatecas Mexico, Pinto) showed the highest antioxidant capacity in the cotyledon, while the Alubia bean, Higuera Azufrado, Negro Criollo (Tulancingo, Coixtlahuaca, Oaxaca, Mexico, black), Bayo (Hidalgotitlan, Veracruz, Mexico, yellow), Cuba-V7, and Negro 8025 (Durango INIFAP, black) varieties showed a lower percent inhibition in this part of the seed.

Finally, when obtaining the average antioxidant capacity from the two parts of the seed analyzed, the Flor de Mayo variety showed the highest values of all varieties evaluated (49.06%), while the Higuera Azufrado variety showed the lowest value (11.46%).

The total phenols seen in the common bean seed coat evaluated ranged from 0.69 mg gallic acid equivalents (GAE)/g to 3.32 mg GAE/g, whereas in the cotyledon these compounds were within the range of 0.44 mg GAE/g to 0.99 mg GAE/g. 

The seed coat showed a greater total phenols content than the cotyledon in all bean varieties evaluated. On average, the total phenols content observed in the seed coat was 1.83 mg GAE/g, while the content of these compounds obtained in the cotyledon was 0.62 mg GAE/g. Similar results were reported by Gálvez et al. [12] in 25 varieties of Brazil beans. These authors reported that condensed tannins, anthocyanins, and flavonols such as kaempferol and quercetin glycosides were mostly found in seed coats. While the cotyledon was rich in phenolic acids, such as ferulic, sinapic, chlorogenic, and other hydroxycinnamic acids.

The Flor de Mayo variety showed the highest content of total phenols in the seed coat, while the variety Higuera Azufrado had the lowest value in this part of the seed. This same behavior was observed in the antioxidant capacity of both varieties, indicating the influence that the phenolic content of the seed coat of the bean has on their antioxidant activity. In accordance with Aguilera et al. [20] antioxidant capacity is directly related to the chemical structure of polyphenols, such as number of hydroxyl groups, degree of glycosylation etc.

In cotyledon the Pinto Saltillo variety had the highest value of total phenols while Negro 8025 variety had the lowest value of these compounds of all the varieties analyzed.

In the present study, a higher content of total phenols in spotted beans than in black beans was observed. Meanwhile the lowest values of total phenols were found in yellow and white bean seeds. This was probably due to the different color of seed coats exhibited by these beans. Other studies have shown similar behavior between pinto and non-colored beans [20]. And, similar behavior was observed in dark and highly pigmented bean varieties [21]. 

Finally, as was observed in the results of antioxidant capacity, when obtaining the average total phenols from the two parts of the seed analyzed, the Flor de Mayo variety showed the highest values of all varieties evaluated (1.95 mg GAE/g), while Higuera Azufrado showed the lowest value (0.63 mg GAE/g). This range of total phenols obtained in the present study is slightly lower than those reported by Espinosa-Alonso et al. [18] in 62 wild and weedy Mexican bean collections from diverse origins.

### 2.2. Measuring Color in Common Bean Varieties

The lightness (L*), a* (red-purple = positive value and green-bluish = negative value) and b* (yellow = positive value and blue = negative value) color coordinates, the hue angle and the chroma of common bean varieties are shown in Table 2. The statistical analysis showed a significant difference (*p* ≤ 0.05) between bean varieties for all color parameters analyzed. The lightness obtained ranged from 23.47 to 77.00.

According to the results obtained, a higher luminance was observed in the navy bean variety (L* = 77.00), which is consistent with the white color of its seed coat. The Negro 8025, Negro San Luis, and Criollo Negro varieties, on the other hand, showed the lowest L* value, having shown no statistical difference, which is consistent with their dark color. The yellow bean, spotted, and Bayo bean varieties showed intermediate lightness values. Their behavior (arranged from greatest to lowest lightness) is shown below: Pinto Saltillo > Higuera Azufrado > Moyocoa > Flor de Mayo > Bayo > Ojo de Cabra beans.

Results obtained are lower than those reported by Rocha-Guzmán et al. [22] in black bean, Pinto Saltillo, and Bayo varieties. The authors indicated that this parameter is significantly affected by thermal processing. However, our results are very similar to those obtained by Aguirre and Gómez-Aldapa [23] with Bayo Victoria, Pinto Saltillo and Negro San Luis bean varieties, who classified the coloration of the first two varieties as reddish yellow, while classifying the Negro San Luis bean variety as greenish blue.

Moreover, the a* color coordinate ranged from 0.78 to 15.46. Given that all values obtained were positive, the trend mainly included reddish shades, being highest in the Flor de Mayo variety, which corresponds to a spotted shade. Likewise, the varieties showing the lowest red coloration trend were the Cuba-V7, the Criollo Negro bean, the Negro San Luis bean and the Negro 8025 bean varieties, showing no statistical difference. Results obtained are higher than those reported by Aguirre and Gómez-Aldapa [23] with the Bayo Victoria, Pinto Saltillo and Negro San Luis bean varieties.

With regard to the b* value, this ranged from −1.82 to 29.60. A number of varieties were observed to show a yellow color trend, since their values were positive, while other varieties showed a blue color trend, since their values were negative. The negative values correspond to dark varieties, such as the Negro San Luis bean, the Negro 8025, Criollo Negro bean, Negro Jamapa bean, and Cuba-V7 varieties, having shown no statistical difference. The other varieties showed a yellow color trend, where the highest value was observed in the Bayo variety, followed by the Moyocoa variety (both consistent with this shade). Other authors have reported negative values for b* in dark seed coat bean varieties, as was the case in this study. Thus, Salinas et al. [14] obtained negative results for the Puebla black bean, Negro 151, Negro 152, Querétaro black bean, Negro San Luis bean, Sinaloa black bean, Veracruz black bean, Medellín bean, Nayarit 80, Jamapa, Mercentral, Altiplano, and Puebla black 152 bean varieties, all of which were classified within the third quadrant of the tristimulus scale, showing a greenish blue coloration. In another study, Aguirre and Gómez-Aldapa [23] also reported a negative value for the Negro San Luis bean variety, which showed a slightly higher value than that obtained in this study.

The chroma defines the spectral intensity or purity of color and ranges from gray, pale and dull shades to more intense and lively shades [24]. This parameter ranged from 0.95 to 36.59 in the bean varieties evaluated. The highest value was shown by the Bayo variety while the lowest one was shown by the Cuba V-7 variety. The chroma value obtained for the Pinto Saltillo variety is similar to that reported by Aguirre and Gómez-Aldapa [23] for this variety; however, these authors found lower results than those obtained in this study for the Bayo Victory and Negro San Luis bean varieties.

With regard to the hue angle, this ranged from 17.91 to 325.65. The dark seed coat varieties (Negro San Luis bean, Criollo Negro bean, Negro Organico bean, Negro 8025, Jamapa and Cuba-V7) were observed to show the highest value in this parameter, which remaining at 301.92–325 and corresponding to blue and purple matiz. On the other hand, the Flor de Mayo variety (from Durango INIFAP, spotted) showed the lowest result (hue angle (°h) = 17.91), followed by the Ojo de Cabra bean (from San Francisco de Conchos, Chihuahua, spotted), Bayo (from Hidalgotitlan, Veracruz, yellow) and the Pinto Saltillo (from Vergelitos Sombrerete, Zacatecas, dotted), corresponding to reddish shades, while the remaining varieties showed yellowish shades (Alubia, Moyocoa and Higuera Azufrado.

### 2.3. Analysis of Phytonutrients Contained in the Seed Coat and Cotyledon of Common Bean

#### 2.3.1. Organic Elements Contained in the Seed Coat and Cotyledon of Common Bean

Table 3 shows the organic elements observed in the seed coat and cotyledon of common bean. 

The statistical analysis showed no effect whatsoever of the seed part (seed coat or cotyledon) on the content of carbon (C) in any of the varieties studied. There was also no significant difference between varieties with regard to carbon concentration in the cotyledon, though the difference was significant in the seed coat, where the Higuera Azufrado variety showed the highest value at 47.24%, and the Flor de Mayo, Alubia and Bayo varieties showed the lowest results, with no statistical difference between them.

Moreover, the statistical analysis showed the effect of bean variety and seed part (*p* ≤ 0.05) both on hydrogen concentration and protein concentration. On average, cotyledon showed a hydrogen content of 6.67%, while the seed coat showed a hydrogen content of 5.73%. With regard to cotyledon, the Pinto Saltillo variety showed the highest concentration at 7.18%, while the Ojo de Cabra variety showed the lowest concentration at 6.05%. Likewise, the highest concentration of hydrogen in seed coat was observed in Moyocoa variety (6.50%), while the lowest concentration in seed coat was seen in the Flor de Mayo variety (4.98%).

With respect to protein, its concentration was higher in cotyledon as compared to seed coat in all varieties studied, having shown a general average of 26.36% and 9.04%, respectively.

There was no significant difference (*p* > 0.05) between bean varieties with regard to protein content in cotyledon, while the seed coat showed the highest concentration in the Higuera Azufrado variety (17.05%), and the Alubia variety showed the lowest concentration (4.57%). Results obtained for cotyledon are similar to those reported by Acosta-Gallego et al. [25] who used various genotypes of common beans grown in Mexico.

In summary, the cotyledon showed a higher hydrogen and protein content than the seed coat. Likewise, the Higuera Azufrado variety (from Guasave, Sinaloa, yellow shade) showed the greatest carbon and protein concentration in the cotyledon, while the Flor de Mayo variety (from Durango INIFAP, spotted) showed the lowest carbon and hydrogen concentration.

#### 2.3.2. Micronutrients Contained in the Seed Coat and Cotyledon of Common Bean Varieties 

Table 4 shows the micronutrient content observed in the seed coat and cotyledon of common bean varieties. The statistical analysis showed significant differences (*p* ≤ 0.05) between bean varieties and between seed parts (cotyledon and seed coat) with regard to all micronutrients evaluated.

On average, cotyledon showed a greater nickel concentration than the seed coat (3.73 ppm and 1.53 ppm, respectively). When performing a test per seed part, the cotyledon in the Bayo bean variety was observed to have the highest nickel concentration (8.82 ppm), while the Flor de Mayo variety showed the lowest concentration (2.20 ppm). Likewise, with regard to the seed coat, the Alubia and Cuba-V7 showed the highest nickel concentrations, while the Criollo Negro variety showed the lowest concentration (0.65 ppm).

Furthermore, the seed coat, on average, showed a higher iron concentration than the cotyledon (91.92 ppm and 47.73 ppm, respectively). The cotyledon in the Higuera Azufrado variety showed the highest iron concentration at 69.06 ppm, while the Criollo Negro variety showed the lowest concentration at 34.17 ppm. With regard to the seed coat, the Cuba-V7 variety had the greatest iron concentration in this seed part (196.15 ppm), while the Flor de Mayo variety had the lowest concentration (21.25 ppm). Results obtained in this study exceeded the results reported by Acosta-Gallego et al. [25] who used various genotypes of common beans grown in Mexico.

With regard to zinc, it showed a higher concentration in the cotyledon as compared to the seed coat (23.53 ppm and 21.49 ppm, respectively). The Bayo variety showed the highest concentration of zinc in the cotyledon (29.82 ppm), while the lowest concentration was observed in the Negro Organico and Negro San Luis bean varieties. The highest zinc concentration in the seed coat was seen in the Moyocoa variety (45.29 ppm), while the Alubia variety showed the lowest concentration (10.49 ppm). In general, the zinc concentration results obtained in this study are slightly lower than the results reported by Acosta-Gallego et al. [25] who used various genotypes of common beans grown in Mexico. These differences may have resulted from the amount of this microelement present in cultivation soils.

With regard to copper, the cotyledon was seen to have a higher concentration than the seed coat. On average, the cotyledon showed a concentration of 9.17 ppm, while the seed coat showed a concentration of 4.60 ppm. Results show that the Moyocoa variety had the highest concentration of this micronutrient, both in the seed coat and the cotyledon, while the Negro 8025, the Negro San Luis bean, and the Flor de Mayo varieties showed the lowest concentration in both seed parts, having shown no significant difference.

Finally, manganese also showed a higher concentration in the cotyledon as compared to the seed coat. The manganese content found in these seed parts was 11.18 ppm and 3.69 ppm, respectively. With regard to cotyledon, the Cuba-V7 variety showed the greatest manganese concentration at 17.02 ppm, while the Flor de Mayo variety showed the lowest concentration of this micronutrient at 8.19 ppm. Likewise, with regard to the seed coat, the Cuba-V7 variety also showed the greatest manganese concentration (9.60), while the Jamapa variety showed the lowest result (2.12 ppm).

In summary, the cotyledon showed the highest concentration of nickel, zinc, copper and manganese, while the seed coat showed the highest concentration of iron. Likewise, the Cuba-V7 variety showed the highest concentration of nickel, iron and manganese in the seed coat of all bean varieties studied. This contrasts the Flor de Mayo variety, which showed the lowest concentration of nickel, copper, and manganese in the cotyledon. According to Bernal et al. [26] soil that has a pH between 6 and 7 is ideal for crops because in this pH range the assimilation of almost all the nutrients by the plants takes place and also the highest biological activity occurs. This could explain the results obtained in the Cuba-V7 variety since the region where it was cultivated presented a soil with a pH very close to the range established as ideal (Table 5), while the Flor de Mayo variety was cultivated in a region with soils with pH levels above that range (Table 5) which did not allow the assimilation of micronutrients in an adequate way by the crop.

The content of micronutrients in the seed coat (descending order) is shown as follows Fe > Zn > Cu > Mn > Ni, while the content of micronutrients in the cotyledon is shown as follows: Fe > Zn > Mn > Cu > Ni.

Similar iron (in cotyledon) and manganese values were reported by Espinosa-García et al. [27] for bean varieties grown in various regions in the state of Oaxaca, Mexico, although the values for copper and zinc were lower than those obtained by these authors. The differences may be due to the effect caused by the genotype, the environment and the genotype interaction per environment, which must be taken into considering when defining goals to enhance these nutritional characteristics [25].

#### 2.3.3. Macronutrient Content in the Seed Coat and Cotyledon of Common Bean Varieties

Table 6 shows the macronutrient content in the seed coat and cotyledon of common bean varieties grown in different parts of Mexico. The statistical analysis showed a significant difference (*p* ≤ 0.05) between bean varieties and between parts of the seed (cotyledon and seed coat) with regard to all macronutrients evaluated.

Cotyledon showed a higher nitrogen content than the seed coat, which on average had a nitrogen content of 4.20% and 1.47%, respectively. No significant difference was observed between bean varieties with regard to cotyledon, though a significant difference was observed in the seed coat of the Higuera Azufrado variety, which showed the highest value at 2.72%, while the Alubia and Negro Organico bean variety showed the lowest concentrations at 0.73% and 0.79%, respectively, having no statistical difference between these two.

Phosphorus also showed a higher concentration in the cotyledon as compared to the seed coat. The phosphorus content found in these seed parts was 0.16% and 0.10%, respectively. The Jamapa variety showed the highest concentration of macronutrients in the cotyledon, while the Negro Organico bean varieties showed the lowest concentration. With regard to the seed coat, the Bayo variety showed the highest value (at 0.26%), whereas the value observed in the Alubia variety was only 0.01%.

Potassium showed an average concentration of 0.50% in the cotyledon, while the concentration seen in the seed coat was 0.25%. The Negro 8025 variety had the highest potassium concentration in the cotyledon (1.02%), while the Negro Organico bean, Flor de Mayo and Pinto Saltillo varieties showed the lowest concentrations. Also, with regard to the seed coat, the Negro San Luis bean showed the highest potassium concentration, whereas the Higuera Azufrado showed the lowest value (0.45% and 0.13%, respectively). Several studies have shown that both genetics and the environmental influence K content of bean seeds [8], which could explain the differences found between the varieties analyzed 

In contrary to other macronutrients, calcium showed a higher concentration in the seed coat (0.96% on average), while the concentration observed in the cotyledon was very low (0.08%). With regard to calcium concentration in the cotyledon, the Ojo de Cabra bean and Negro Organico bean varieties showed the highest concentration of this macronutrient, whereas the Criollo Negro bean and Negro San Luis bean varieties showed the lowest concentrations. With regard to the seed coat, the highest calcium concentrations were seen in the Alubia and Negro Organico bean varieties (1.36% and 1.34%, respectively), while the lowest value was seen in the Higuera Azufrado variety (0.49%). Results obtained in this study far exceed the results reported by Acosta-Gallego et al. [23] who used various genotypes of Mexican common bean, including the Pinto Saltillo, Flor de Mayo, and Higuera Azufrado varieties, which were also tested in this study.

Magnesium also showed a higher concentration in the seed coat as compared to the cotyledon, with 0.30% and 0.09%, respectively. With regard to the seed coat, the Negro San Luis bean, Alubia, Criollo Negro bean, Negro 8025, Negro Organico bean, and Higuera Azufrado varieties showed the greatest concentration of this macronutrient, having shown no significant difference between these varieties, while the Cuba-V7 variety showed the lowest result. Likewise, with regard to cotyledon, the Alubia and Ojo de Cabra bean varieties showed the highest concentration (at 0.11% each). There was no statistical difference between the two varieties. The Jamapa variety, however, showed the lowest concentration at 0.06%.

Sulfur also showed a higher concentration in the cotyledon as compared to the seed coat (0.12% and 0.08%, respectively). The Negro 8025 variety showed the highest sulfur concentration found in the cotyledon, while the Alubia, Negro Organico bean, Bayo, and Cuba-V7 varieties showed the lowest concentrations. There was no statistical difference between the latter. With regard to the seed coat, the Higuera Azufrado variety showed the highest sulfur concentration, while no content of this macronutrient was found in the Flor de Mayo variety.

The variation in the mineral content between the different varieties analyzed in the present investigation can be attributed to the genotype and environment in which they were produced (Table 5) since these factors have a great influence on the nutritional content of the common bean [28]. In summary, the cotyledon showed a higher concentration of nitrogen, phosphorus, potassium and sulfur as compared to the seed coat, whereas the seed coat showed a higher concentration of calcium and magnesium. With regard to the seed coat, the Higuera Azufrado variety contained a higher concentration of nitrogen and sulfur, though a lower concentration of potassium and calcium.

The content of macronutrients in the seed coat is shown as follows (descending order): N > K > S > P > Mg > Ca, whereas in the case of cotyledon, the content is as follows: N > Ca > Mg > K > P > S.

The macronutrient content observed in this study was much lower than that reported by Espinosa-García et al. [27] for bean varieties grown in various regions in the state of Oaxaca, Mexico.

### 2.4. Correlation Analysis between Variables

Table 7 shows the Pearson correlation coefficients for the general correlation analysis performed with all variables evaluated using the bean samples studied.

Lightness showed no significant correlation with any of the phytonutrients analyzed, though it did show a highly significant correlation with the b* color coordinate and the hue angle. The relationship with the former was positive (*r* = 0.74) while the relationship with the latter was negative (*r* = −0.88). Moreover, the a* color coordinate showed a significant correlation with phosphorus (*r* = 0.22) and zinc (*r* = 0.24), and a highly significant correlation with iron (*r* = −0.44). Moreover, the value of b* was significantly correlated with phosphorus (*r* = 0.32), nickel (*r* = 0.23), iron (*r* = −0.23) and copper (*r* = 0.41), also showing a highly significant correlation with zinc (*r* = 0.63). With respect to the chroma, it did not show a significant correlation with any of the variables tested, while the hue angle showed a positive significant correlation with iron (*r* = 0.36) and a negative correlation with zinc (*r* = −0.36). In contrary to these results, Kahraman and Onder [16] did not find a significant correlation between color and mineral content in 39 common bean genotypes widely grown in Turkey.

On the other hand, a highly significant negative correlation was observed between antioxidant capacity and nickel (*r* = −0.63), copper (*r* = −0.68), manganese (*r* = −0.80), potassium (*r* = −0.43), hydrogen (*r* = −0.50) and protein (*r* = −0.76), while a highly significant positive correlation was seen between antioxidant capacity and calcium (*r* = 0.72) and magnesium (*r* = 0.78). According to some authors, the mineral elements maintain a relationship with the antioxidant activity of plants. The structure of antioxidant enzymes also contains the corresponding metal, and the antioxidant process requires the participation of some metal ions as well. Copper, zinc, iron, and manganese are the ingredients of some of the important antioxidant enzymes. For example, copper, zinc, iron, and manganese are components of Cu/Zn-SOD (superoxide dismutase), Fe-SOD and Mn-SOD. These metals are important components of the enzyme structure. Some are involved in the expression of the enzyme activity, some are the enzyme activity material, and adequacy and deletion of these metal elements will affect the enzyme activities, and also affect the antioxidant capacity of the enzyme system. The antioxidant enzymes activities are correlated with biological electron transfer processes. Due to the fact that the properties of the transition metals Cu, Zn, Fe, and Mn easily gain and lose electrons, these metals are the important external factors which affect the occurrence, transfer, and loss of reactive oxygen species (ROS), as well as the mutual influence and transformation [29]. 

Likewise, a highly significant positive correlation was observed between the content of total phenols and antioxidant capacity (*r* = 0.88), magnesium (*r* = 0.68) and calcium (*r* = 0.66) and there was a significant negative correlation between total phenols and phosphorus (*r* = −0.28), nickel (*r* = −0.52), copper (*r* = −0.57), manganese (*r* = −0.69), potassium (*r* = −0.32), nitrogen (*r* = −0.68), hydrogen (*r* = −0.47), sulfur (*r* = −0.25) and protein (*r* = −0.68).

The high correlation observed between total phenols and antioxidant activity was very similar to that reported by Mastura et al. [30] in eight types of Malaysian beans. In another study, Aquino-Bolaños et al. [31] also observed that the antioxidant capacity was significantly correlated (*r* > 0.36) with total polyphenol content in the seed coat and in the whole seed in samples of 26 populations of common bean which were collected in various rural communities in the states of Oaxaca, Guerrero, Puebla, Tlaxcala and Estado de Mexico, Mexico.

The results of the present investigation indicate an important contribution of the content of total phenols with the antioxidant activity of Mexican beans. Thus, total phenolic content could be used as an indicator in evaluating the antioxidant capacity of beans which may preliminarily applied as natural sources of antioxidant functional foods [32]. Polyphenols are natural antioxidants scavenging free radicals, binding transition metal ions (Fe^2+^ and Cu^2+^), and preventing, lipid peroxidation [33]. On the other hand, in the present investigation, a significant correlation was found between total phenols and some minerals; similar results were observed by Grela et al. [33] who reported that total phenols were significantly correlated with the content of K (white lupine (+)), Na (Andean lupine (−) and grass pea (+)), P (pea and lentil (+)), Ca (yellow and Andean lupines (+)), Mg (white and yellow lupines, pea, and lentil (+)), Fe (yellow lupine (+)), and Cu (broad bean (+)).

## 3. Materials and Methods 

### 3.1. Bean Variety Collection

The bean varieties collected, including their place of origin, color, pH of soil and climate of the place where they were cultivated are shown in Table 5.

Bean varieties were selected based on their seed coat color, producing region and consumption popularity.

### 3.2. Sample Preparation

Bean seeds were harvested in 2017 and collected and analyzed in 2018. This study used 100 seeds from each bean variety evaluated. The seed coat and cotyledon were carefully removed using a scalpel until obtaining a 14 g sample size for each component. Afterwards, the samples were ground and stored in polyethylene bags, which were kept in desiccators until testing. From the total sample, 10 g were used to analyze the antioxidant capacity while the remaining 4 g were used to determine the content of minerals (N, P, K, Ca, Mg, Fe, Mn, Zn, Cu, Ni), organic elements (C, H, S), and proteins.

### 3.3. Measuring the Antioxidant Capacity (DPPH) and Total Phenols

Antioxidant activity was measured using the 2,2-diphenyl-1-picrylhydrazyl (DPPH) method according to the methodology applied by Hsu et al. [34]. The methanolic extract was obtained by soaking 1 g of seeds in 5 mL of 80% methanol. The resulting mixture was centrifuged at 6000× *g* for 10 min, taking 0.5 mL of the resulting supernatant from extract to mix it with 2.5 mL of freshly prepared 0.1 mM DPPH solution. The mixture was incubated for 60 min in darkness and cold conditions. Absorbance was measured by spectrophotometry at 517 nm. For the blank solution, the extract was replaced with 0.5 mL of methanol. The test values for DPPH were obtained by using the following formula:

Scavenging effect (%) = (1 − (A517 sample/A517 blank)) (100).(1)

The measurement and quantification of total phenols was determined as suggested by Singleton and Rossi [35]; Singleton et al. [36]. We began by standardizing 0.5–1 g of ground seeds with 5 mL of methanol and 2.5 mL of 1% NaCL solution. The reaction mixture consisted in placing 750 mL of 2% Na_2_CO_3_, 250 mL of 50% Folin—Ciocalteau reagent and 1375 mL of deionized water in a test tube, adding 250 mL of extract. The mixture was incubated at room temperature for 60 min, after which the measurement was done against a gallic acid (10–100 mg/mL) pattern curve at 725 nm absorbance. Results are shown in mg GAE/g dry weight (dw).

For the analysis of the antioxidant capacity and total phenols three repetitions were used

### 3.4. Color Measurement

This variable was measured using a portable Konica Minolta DP-400 colorimeter (Minolta Co. Ltd., Osaka, Japan). The equipment was used to obtain (CIE: Commission Internationale d’Eclairage) (LAB = L*, a* and b*) CIELAB system coordinates (International Commission on Illumination). The "L” parameter represents luminance, which ranges from 0 (black) to 100 (white); a* can have either positive (red) or negative (green) values and b* ranges from yellow when the value is positive to blue when the value is negative. Color measurements were made in triplicate on the surface of the bean. The test used a sample of 50 seeds, which were placed in a Petri dish to determine color.

The L*, a*, and b* coordinates were used to obtain the CIEL*C*h° color space, where C* represents the chroma or color saturation and h° is the hue angle or hue representing the shade according to the angle in the 360° color wheel, with a red-purple shade at 0°, a yellow shade at 90°, a gray-green shade at 180° and a blue shade at 270°, counterclockwise [37].

The chroma and hue were calculated using the following formulas [37]:

C∗ = (a∗^2^ + b∗^2^)^1/2^

°h = 360° + [(arctan (b*/a*))/6.2832]*360

When a* > 0 and b* < 0

Or 

°h = [(arctan (b*/a*))/6.2832]*360

When a > 0 and b > 0

### 3.5. Phytonutrient Test

#### 3.5.1. Measurement of Organic Compounds (Carbon, Hydrogen and Protein), Nitrogen and Sulfur Content in Seed Coat and Cotyledon in Common Bean Varieties

Here, 3 µg of the sample were weighted in a nickel capsule, adding 9 µg of vanadium pentoxide (V_2_O_5_). The mixture was then introduced in the Flash 2000 device (Thermo Scientific, Cambridge, UK), which works according to the Dumas method [38]. Concentrations are shown as a percentage. For the analysis three repetitions were used.

#### 3.5.2. Measuring Fe, Zn, Na, Mg, Mn, K, Ca, Cu and Ni Content

To measure the concentration of minerals (K, Ca, Mg, Fe, Mn, Zn, Cu, Ni), the samples were ground in a small cup blender (Osterizer^®^ all-metal drive. Boca Ratón, FL, USA) until the grind up point was reached, and the material was transferred to plastic bags (Nasco Whirl-Pak^®^, Fort Atkinson Jefferson County, WI, USA), a 1 g sample of both the seed coat and cotyledon was previously digested using a triacid mixture containing pure nitric acid, pure hydrochloric acid, and sulfuric acid according to a ratio of 1:0.1:0.4 (*v/v/v*) [39], in a digester (Labconco^®^, Kansas City, MO, USA) for 90 min at a temperature of 150 °C, to eliminate organic matter and obtain the mineral fraction [40]. The concentration of these minerals was measured by atomic absorption spectrophotometry (Thermo Scientific, Cambridge, UK) and is expressed in ppm for micronutrients and percentage for macronutrients.

For the analysis three repetitions were used.

#### 3.5.3. Phosphorus Measurement

The phosphorus concentration measurement was performed using the ammonium metavanadate (NH_4_VO_3_) method and the visible light spectrophotometry method (Jenway Spectrophotometer). 0.5 mL of each sample were placed in a test tube, adding 1 mL of ammonium molybdate ((NH_4_)_6_Mo_7_O_24_·4H_2_O) and 3.5 mL of triple distilled water. The samples were mixed using a vortex mixer (VWR), letting them rest for an hour afterwards. After one hour had elapsed, readings were taken for each sample. The phosphorus concentration is shown as a percentage [41]. For the analysis three repetitions were used.

### 3.6. Data Analysis

Data were analyzed using a variance analysis to evaluate the effect that bean variety and the type of seed part (seed coat or cotyledon) has on antioxidant capacity, color and content of macronutrients and micronutrients of beans using the Statistic Analysis Software (SAS) (SAS Institute Inc., Cary, NC, USA). The comparison of means was done with the Tukey test using the same statistical suite. Means were accepted as significantly different to a 95% confidence interval (*p* ≤ 0.05). Results are reported as mean ± standard deviation. A correlation analysis was also done between variables using the SAS statistical suite at a significance level of (*p* ≤ 0.05).

## 4. Conclusions

This study showed the bean genotype and the seed part (cotyledon or seed coat) have a significant effect on the color, antioxidant capacity and phytonutrient content of bean grains.

The values obtained for the color parameter were consistent with the values obtained for coloration for the seeds tested. Thus, the highest luminance was observed in lighter shade varieties, such as the navy bean variety, while the lowest luminance was observed with regard to the seed coat of darker shade varieties, such as the black 8025, San Luis black bean and creole black varieties. The same result was seen for antioxidant capacity, where all dark seed coat varieties showed the highest results.

Cotyledon showed a higher content of protein, H, Ni, Zn, Cu, N, P, K S and Mn, while the seed coat showed a higher content of Fe, Ca, and Mg and a greater antioxidant capacity.

The varieties that stood out in the seed coat test were: the Cuba-V7, which showed the highest concentration of nickel, iron and manganese; the black-eyed beans, Negro 8025, Flor de Mayo and Negro San Luis bean varieties, which showed the greatest antioxidant capacity; the Higuera Azufrado variety, which showed the highest protein and carbon concentration; and the Moyocoa variety, which showed the highest zinc and copper concentration. No variety stood out with regard to protein and carbon content in the cotyledon. The Pinto Saltillo showed the highest antioxidant capacity and hydrogen concentration in the cotyledon, the Bayo variety showed the highest nickel and zinc concentration, the Higuera Azufrado showed the highest iron concentration and the Moyocoa variety showed the highest copper concentration.

Research suggests that in genetic enhancement programs focusing on protein content, the concentration of phytonutrients, such as phosphorus, nickel, copper, manganese, carbon, hydrogen and sulfur could be taken into consideration to increase the ratio of this nutrients in common bean varieties due to their highly significant direct correlation. The same holds true for calcium and magnesium, which can improve antioxidant capacity. 

It is important to characterize Mexican common bean varieties with regard to their content of valuable phytonutrients such as iron, as well as their protein content and their antioxidant capacity. This information is a very valuable tool when designing genetic enhancement programs or when implementing biofortification strategies in order to make up for the needs of these nutrients among the population.

## Figures and Tables

**Table 1:** Antioxidant capacity (% inhibition) and total phenols (mg gallic acid equivalents (GAE)/g) observed in seed coat and cotyledon of common bean varieties.

Variety	Antioxidant Capacity		Total Phenols	
	Seed Coat	Cotyledon	Seed Coat	Cotyledon
Negro 8025	^a^81.60 ± 3.23^A^	^ef^1.43 ± 0.13^B^	^d^2.47 ± 0.00^A^	^m^0.44 ± 0.00^B^
Flor de Mayo	^a^82.18 ± 4.80 ^A^	^b^15.94 ± 1.38^B^	^a^3.32 ± 0.00^A^	^h^0.59 ± 0.00^B^
Negro San Luis	^a^79.42 ± 6.85^A^	^c^13.86 ± 0.47^B^	^f^1.86 ± 0.00^A^	^g^0.60 ± 0.00^B^
Higuera Azufrado	^e^23.86 ± 0.58^A^	^ef^1.29 ± 0.15^B^	^m^0.69 ± 0.00^A^	^i^0.57 ± 0.00^B^
Moyocoa	^d^38.66 ± 0.58^A^	^f^1.19 ± 0.51^B^	^j^1.07 ± 0.00^A^	^f^0.61 ± 0.00^B^
Negro Criollo	^a^78.39 ± 0.59^A^	^f^1.056 ± 0.03^B^	^g^1.86 ± 0.00^A^	^k^0.51 ± 0.00^B^
Ojo de Cabra	^a^84.10 ± 1.47^A^	^e^3.17 ± 0.20^B^	^b^2.93 ± 0.00^A^	^b^0.83 ± 0.00^B^
Cuba-V7	^e^27.56 ± 3.08^A^	^ef^1.535 ± 0.12^B^	^k^0.99 ± 0.00^A^	^l^0.45 ± 0.00^B^
Pinto Saltillo	^c^50.06 ± 8.45^A^	^a^29.77 ± 1.40^B^	^c^2.67 ± 0.00^A^	^a^0.99 ± 0.00^B^
Negro Orgánico	^c^48.80 ± 3.39	^d^8.87 ± 0.75^B^	^e^2.12 ± 0.00^A^	^d^0.69 ± 0.00^B^
Negro Jamapa	^b^60.94 ± 1.00^A^	^d^9.18 ± 2.87^B^	^i^1.24 ± 0.00^A^	^c^0.72 ± 0.00^B^
Bayo	^a^75.492 ± 6.83^A^	^ef^1.37 ± 0.33^B^	^h^1.65 ± 0.00^A^	^e^0.61 ± 0.00^B^
Alubia	^e^28.75 ± 0.78^A^	^f^0.667 ± 0.11^B^	^l^0.99 ± 0.00^A^	^j^0.53 ± 0.00^B^

The means shown in different capital letters per line indicate a significant statistical difference (*p* ≤ 0.05) between the seed coat and cotyledon within each variety. The means shown in lowercase letters per column indicate a significant statistical difference (*p* ≤ 0.05) between bean varieties within each part of the seed (seed coat and cotyledon). Mean ± Standard Deviation.

**Table 2:** Lightness (L*), a* (red-purple) and b* (yellow-purple) color coordinates, hue angle and chroma value observed in common bean varieties.

Variety	L*	a*	b*	Chroma	Hue Angle
Negro 8025	23.60 ± 0.335^h^	1.153 ± 0.130^gh^	−1.72 ± 0.460^i^	2.07 ± 0.435^g^	304.60 ± 5.80^b^
Flor de Mayo	51.52 ± 0.935^e^	15.46 ± 0.183^a^	4.99 ± 0.44^g^	16.25 ± 0.146^e^	17.91 ± 1.67^f^
Negro San Luis	23.47 ± 0.147^h^	1.136 ± 0.066^gh^	−1.82 ± 0.060^i^	2.14 ± 0.085^g^	301.92 ± 0.73^b^
Higuera Azufrado	63.89 ± 0.680^c^	2.05 ± 0.196^f^	28.48 ± 0.085^c^	28.56 ± 0.071^c^	85.87 ± 0.40^c^
Moyocoa	60.98 ± 0.77^d^	5.206 ± 0.162^d^	29.60 ± 0.508^b^	30.06 ± 0.522^b^	80.02 ± 0.210^c^
Negro Criollo	23.51 ± 0.958^h^	0.94 ± 0.030^gh^	−1.44 ± 0.344^i^	1.73 ± 0.277^gh^	303.82 ± 6.17^b^
Ojo de Cabra	48.32 ± 0.696^f^	7.16 ± 0.061^b^	13.93 ± 0.168^e^	15.66 ± 0.177^e^	62.78 ± 0.100^e^
Cuba-V7	24.95 ± 0.506^hg^	0.78 ± 0.06^h^	−0.54 ± 0.09^h^	0.95 ± 0.080^h^	325.65 ± 4.36^a^
Pinto Saltillo	68.94 ± 0.634^b^	6.03 ± 0.151^c^	6.03 ± 0.151^d^	18.18 ± 0.279^d^	70.60 ± 0.75^de^
Negro Orgánico	26.23 ± 0.232^g^	1.19 ± 0.060^g^	1.19 ± 0.060^i^	2.14 ± 0.125^g^	303.79 ± 1.43^b^
Negro Jamapa	26.22 ± 0.381^g^	1.21 ± 0.170^g^	−1.59 ± 0.05^i^	2.00 ± 0.075^g^	307.27 ± 4.64^b^
Bayo	48.45 ± 0.83^f^	15.14 ± 0.145^a^	33.31 ± 0.41^a^	36.59 ± 0.400^a^	65.55 ± 0.29^e^
Alubia	77.00 ± 1.685^a^	2.47 ± 0.034^e^	2.47 ± 0.034^f^	12.68 ± 0.203^f^	78.76 ± 0.074^cd^

The means shown in different letters per column indicate a significant statistical difference (*p* ≤ 0.05) between bean varieties. Mean ± Standard Deviation.

**Table 3:** Organic elements contained in the seed coat and cotyledon of common bean varieties.

Variety	C		H		Protein	
	Cotyledon	Seed Coat	Cotyledon	Seed Coat	Cotyledon	Seed Coat
Negro 8025	^a^43.43 ± 2.22^A^	^abc^41.72 ± 1.34^A^	^ab^6.87 ± 0.40^A^	^abcde^5.85 ± 0.36^B^	^a^27.09 ± 1.06^A^	^bcd^7.37 ± 2.09^B^
Flor de Mayo	^a^43.94 ± 1.74^A^	^c^35.48 ± 6.24^A^	^ab^6.92 ± 0.32^A^	^e^4.98 ± 0.99^B^	^a^26.82 ± 1.45^A^	^cd^6.23 ± 2.42^B^
Negro San Luis	^a^43.54 ± 1.78^A^	^abc^43.08 ± 0.87^A^	^ab^6.93 ± 0.33^A^	^abcde^5.80 ± 0.32^B^	^a^26.45 ± 2.11^A^	^abcd^9.69 ± 7.84^B^
Higuera Azufrado	^a^43.44 ± 1.26^A^	^a^47.24 ± 11.65^A^	^ab^6.88 ± 0.18^A^	^ab^6.40 ± 0.96^A^	^a^26.68 ± 3.54^A^	^a^17.05 ± 9.79^B^
Moyocoa	^a^41.53 ± 2.76^A^	^abc^42.83 ± 4.70^A^	^ab^6.60 ± 0.47^A^	^a^6.50 ± 0.87^A^	^a^26.49 ± 2.13^A^	^abc^13.92 ± 7.22^B^
Negro Criollo	^a^42.37 ± 0.95^A^	^bc^38.90 ± 1.93^B^	^ab^6.72 ± 0.23^A^	^bcde^5.38 ± 0.27^B^	^a^28.38 ± 1.27^A^	^cd^5.87 ± 0.36^B^
Ojo de Cabra	^a^38.90 ± 6.55^A^	^ab^44.59 ± 6.21^A^	^b^6.05 ± 1.03^A^	^abc^6.35 ± 1.10^A^	^a^28.52 ± 5.98^A^	^ab^15.15 ± 7.80^B^
Cuba-V7	^a^41.09 ± 3.88^A^	^bc^39.34 ± 2.790^A^	^ab^6.47 ± 0.65^A^	^cde^5.33 ± 0.41^A^	^a^24.12 ± 2.28^A^	^bcd^7.34 ± 2.89^B^
Pinto Saltillo	^a^45.01 ± 6.45^A^	^abc^41.59 ± 2.71^A^	^a^7.18 ± 1.09^A^	^abcd^6.03 ± 0.46^A^	^a^28.62 ± 4.06^A^	^cd^6.57 ± 0.76^B^
Negro Orgánico	^a^42.98 ± 5.99^A^	^bc^39.17 ± 2.09^A^	^ab^6.69 ± 0.76^A^	^cde^5.36 ± 0.24^B^	^a^23.70 ± 3.13^A^	^d^4.95 ± 1.08^B^
Negro Jamapa	^a^41.36 ± 1.11^A^	^abc^42.62 ± 1.19^A^	^ab^6.42 ± 0.18^A^	^abcde^5.98 ± 0.10^B^	^a^24.14 ± 0.95^A^	^abcd^12.03 ± 3.51^B^
Bayo	^a^41.28 ± 7.20^A^	^c^38.40 ± 1.73^A^	^ab^6.57 ± 1.180^A^	^e^5.19 ± 0.37^A^	^a^25.30 ± 5.50^A^	^bcd^6.81 ± 4.75^B^
Alubia	^a^42.34 ± 4.37^A^	^c^36.08 ± 1.98^A^	^ab^6.36 ± 0.59^A^	^e^5.28 ± 0.34^A^	^a^25.56 ± 2.81^A^	^d^4.57 ± 0.97^B^

The means shown in different capital letters per line indicate a significant statistical difference (*p* ≤ 0.05) between the seed coat and cotyledon within each variety. The means shown in different lowercase letters per column indicate a significant statistical difference (*p* ≤ 0.05) between bean varieties within each part of the seed. Mean ± Standard Deviation.

**Table 4:** Micronutrients content (ppm) in the seed coat and cotyledon of common bean varieties.

**Variety**	**Ni**		**Fe**		**Zn**	
	**Cotyledon**	**Seed Coat**	**Cotyledon**	**Seed Coat**	**Cotyledon**	**Seed Coat**
Negro 8025	^de^2.99 ± 0.17^A^	^dcefg^1.32 ± 0.49^B^	^bc^52.67 ± 0.48^B^	^efg^78.28± 15.35^A^	^def^21.51 ± 0.88^A^	^ef^13.51 ± 1.99^B^
Flor de Mayo	^e^2.20 ± 0.89^A^	^efg^0.90 ± 0.17^A^	^cdef^48.16 ± 2.33^A^	^h^21.2 5 ± 1.66^B^	^efg^19.38 ± 0.86^A^	^ef^12.47 ± 0.52^B^
Negro San Luis	^de^2.83 ± 0.47^A^	^bcdefg^1.38 ± 0.54^B^	^efg^43.41 ± 1.42^B^	^cde^94.75 ± 2.16^A^	^g^16.31 ± 1.85^A^	^ef^12.91 ± 1.00^B^
Higuera Azufrado	^bc^4.27 ± 0.32^A^	^defg^1.14 ± 0.52^B^	^a^69.06 ± 1.84^B^	^cde^94.89 ± 3.51^A^	^cde^24.13 ± 1.26^A^	^c^26.88 ± 3.59^A^
Moyocoa	^de^2.78 ± 0.39^A^	^fg^0.82 ± 0.14^B^	^cde^48.52 ± 6.53^B^	^g^71.26 ± 3.77^A^	^abc^27.87 ± 0.33^B^	^a^45.29 ± 3.72^A^
Negro Criollo	^bcd^3.67 ± 0.65^A^	^g^0.65 ± 0.54^B^	^h^34.17 ± 1.44^B^	^def^91.86 ± 4.41^A^	^bcd^25.05 ± 6.94^A^	^d^18.46 ± 0.63^A^
Ojo de Cabra	^de^3.09 ± 0.72^A^	^abcdef^1.54 ± 0.13^B^	^gh^38.47 ± 1.53^B^	^fg^75.79 ± 0.26^A^	^ab^29.27 ± 2.70^A^	^c^26.85 ± 2.66^A^
Cuba-V7	^d^3.28 ± 0.70^A^	^a^2.27 ± 0.47^A^	^b^57.96 ± 8.83^B^	^a^196.15 ± 33.43^A^	^abcd^25.69 ± 1.30^A^	^de^16.09 ± 1.50^B^
Pinto Saltillo	^e^2.93 ± 0.12^A^	^abcde^1.66 ± 0.65^B^	^efg^42.90 ± 1.68^B^	^fg^75.62 ± 5.50^A^	^ab^29.30 ± 6.48^A^	^b^33.74 ± 3.77^A^
Negro Orgánico	^cd^3.62 ± 0.25^A^	^abc^2.07 ± 0.56^B^	^ef^45.34 ± 1.83^B^	^b^156.56 ± 3.34^A^	^g^15.82 ± 1.62^A^	^ef^13.91 ± 1.02^A^
Negro Jamapa	^cd^3.60 ± 0.27^A^	^abcd^1.71 ± 0.06^B^	^fg^42.35 ± 2.16^B^	^c^110.79 ± 10.51^A^	^cd^24.48 ± 1.24^A^	^d^19.29 ± 0.74^B^
Bayo	^a^8.62 ± 0.45^A^	^ab^2.15 ± 0.80^B^	^def^46.25 ± 3.42^A^	^h^27.69 ± 2.14^B^	^a^29.82 ± 1.13^A^	^c^29.46 ± 2.06^A^
Alubia	^b^4.55 ± 0.89^A^	^a^2.21 ± 0.31^B^	^cd^51.29 ± 3.56^B^	^cd^100.09 ± 3.56^A^	^fg^17.26 ± 2.26^A^	^f^10.49 ± 0.78^B^
**Variety**	**Cu**		**Mn**			
	**Cotyledon**	**Seed Coat**	**Cotyledon**	**Seed Coat**		
Negro 8025	^f^6.51 ± 0.15^A^	^d^1.93 ± 0.53^B^	^a^16.46 ± 1.10^A^	^efgh^2.28 ± 0.17^B^		
Flor de Mayo	^f^6.13 ± 0.60^A^	^d^2.44 ± 0.14^B^	^fg^8.19 ± 0.55^A^	^efg^2.99 ± 0.19^B^		
Negro San Luis	^f^5.93 ± 0.31A	^d^1.82 ± 0.47^B^	^efg^8.84 ± 0.54^A^	^efg^2.46 ± 0.21^B^		
Higuera Azufrado	^b^11.25 ± 0.87^A^	^bc^4.83 ± 0.52^B^	^bc^12.60 ± 0.67^A^	^b^5.88 ± 1.40^B^		
Moyocoa	^a^13.06 ± 0.67^A^	^a^7.34 ± 0.95^B^	^cdef^10.53 ± 1.38^A^	^cd^4.33 ± 0.15^B^		
Negro Criollo	^bc^10.66 ± 0.98^A^	^bc^5.14 ± 0.68^B^	^g^7.15 ± 0.32^A^	^h^1.18 ± 0.08^B^		
Ojo de Cabra	^de^8.91 ± 0.97^A^	^ab^6.69 ± 0.95^B^	^def^10.24 ± 0.81^A^	^bc^5.12 ± 1.23^B^		
Cuba-V7	^bc^10.63 ± 0.95^A^	^bc^5.24 ± 3.58^A^	^a^17.02 ± 1.01^A^	^a^9.60 ± 0.97^B^		
Pinto Saltillo	^bcd^10.18 ± 0.42^A^	^ab^5.97 ± 0.86^B^	^efg^9.19 ± 2.66^A^	^efg^2.49 ± 0.13^B^		
Negro Orgánico	^f^6.95 ± 0.42^A^	^bc^4.86 ± 0.77^B^	^defg^9.22 ± 0.66^A^	^def^3.34 ± 0.11^B^		
Negro Jamapa	^cde^9.51 ± 0.27^A^	^cd^3.41 ± 0.27^B^	^bcde^10.95 ± 0.65^A^	^gh^2.12 ± 0.79^B^		
Bayo	^b^11.19 ± 1.47^A^	^ab^6.58 ± 0.20^B^	^bcd^11.64 ± 0.63^A^	^efg^2.79 ± 0.34^B^		
Alubia	^e^8.30 ± 0.74^A^	^cd^3.56 ± 0.16^B^	^b^13.37 ± 3.72^A^	^de^3.42 ± 0.69^B^		

The means shown in different capital letters per line indicate a significant statistical difference (*p* ≤ 0.05) between the seed coat and cotyledon within each variety. The means shown in lowercase letters per column indicate a significant statistical difference (*p* ≤ 0.05) between bean varieties within each part of the seed. Mean ± Standard Deviation.

**Table 5:** Common bean varieties, place of origin, color of seeds, soil pH and climate of the region of origin.

Variety	Place of Origin	Color	Picture	Soil pH	Climate
Negro 8025	Durango INIFAP	Black bean	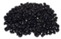	8.15	Semi-arid temperate
Flor de Mayo	Durango INIFAP	Spotted	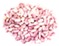	8.15	Semi-arid temperate
Negro San Luis	Durango INIFAP	Black bean	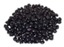	8.15	Semi-arid temperate
Higuera Azufrado	Guasave, Sinaloa	Yellow	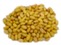	6.6	Very warm semi-dry
Moyocoa	Guasave, Sinaloa	Yellow	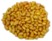	6.6	Very warm semi-dry
Negro Criollo	Tulancingo, Coixtlahuaca, Oaxaca	Black bean	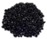	6.5	Dry semi-warm
Ojo de Cabra	San Francisco de Conchos, Chihuahua	Spotted	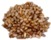	7.21	Extreme semiarid
Cuba-V7	San Jose Las Lajas, Cuba	Black bean	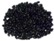	5.5	Tropical subhumid
Pinto Saltillo	Vergelitos Sombrerete, Zacatecas	Pinto	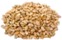	7.5	Temperate semi-dry
Negro Orgánico	Puebla	Black bean	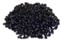	6.9	Tempering subhumid
Negro Jamapa	Teapa, Tabasco	Black bean	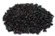	5.18	Tropical wet
Bayo	Hidalgotitlan, Veracruz	Yellow	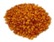	6.0	Warm wet
Alubia	Guadalajara, Jalisco	White	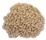	6.48	Temperate semi-dry

INIFAP: Instituto Nacional de Investigaciones Forestales Agricolas y Pecuarias (National Institute for Forest, Agricultural and Cattle Industry Research).

**Table 6:** Macronutrient content (%) in the seed coat and cotyledon of common bean varieties.

**Variety**	**N**		**P**		**K**	
	**Cotyledon**	**Seed Coat**	**Cotyledon**	**Seed Coat**	**Cotyledon**	**Seed Coat**
Negro 8025	^a^4.33 ± 0.16^A^	^bcd^1.18 ± 0.33^B^	^bcd^0.15 ± 0.03^A^	^de^0.08 ± 0.001^B^	^a^1.02 ± 0.27^A^	^cd^0.17 ± 0.03^B^
Flor de Mayo	^a^4.29 ± 0.23^A^	^cd^0.99 ± 0.38^B^	^d^0.10 ± 0.08^A^	^b^0.16 ± 0.01^A^	^e^0.21 ± 0.04^B^	^ab^0.39 ± 0.09^A^
Negro San Luis	^a^4.23 ± 0.33^A^	^abcd^1.55 ± 1.25^A^	^abc^0.20 ± 0.02^A^	^e^0.077 ± 0.007^B^	^cde^0.39 ± 0.09^A^	^a^0.45 ± 0.29^A^
Higuera Azufrado	^a^4.96 ± 0.56^A^	^a^2.72 ± 1.56^B^	^bc^0.19 ± 0.009^A^	^c^0.13 ± 0.02^B^	^ab^0.72 ± 0.14^A^	^d^0.13 ± 0.03^B^
Moyocoa	^a^4.23 ± 0.34^A^	^abc^2.22 ± 1.15^B^	^abc^0.21 ± 0.07^A^	^bc^0.15 ± 0.002^A^	^ab^0.85 ± 0.54^A^	^abc^0.31 ± 0.05^A^
Negro Criollo	^a^4.54 ± 0.20^A^	^cd^0.93 ± 0.05^B^	^bcd^0.15 ± 0.01^A^	^g^0.04 ± 0.023^B^	^e^0.22 ± 0.04^A^	^abcd^0.29 ± 0.06^A^
Ojo de Cabra	^a^4.56 ± 0.95^A^	^ab^2.42 ± 1.24^B^	^cde^0.13 ± 0.08^A^	^bc^0.146 ± 0.018^A^	^e^0.36 ± 0.076^A^	^cd^0.210 ± 0.166^A^
Cuba-V7	^a^3.86 ± 0.36^A^	^bcd^1.17 ± 0.46^B^	^ab^0.23 ± 0.01^A^	^ef^0.07 ± 0.006^B^	^abcd^0.71 ± 0.15^A^	^cd^0.15 ± 0.03^B^
Pinto Saltillo	^a^4.58 ± 0.65^A^	^cd^1.05 ± 0.12^B^	^abc^0.20 ± 0.03^A^	^ef^0.05 ± 0.008^B^	^e^0.23 ± 0.10^A^	^cd^0.19 ± 0.04^A^
Negro Orgánico	^a^3.79 ± 0.50^A^	^d^0.79 ± 0.17^B^	^e^0.050 ± 0.016^A^	^gh^0.036 ± 0.004^A^	^e^0.20 ± 0.04^B^	^ab^0.39 ± 0.04^A^
Negro Jamapa	^a^3.86 ± 0.15^A^	^abcd^1.92 ± 0.56^B^	^a^0.24 ± 0.01^A^	^d^0.10 ± 0.005^B^	^de^0.38 ± 0.06^A^	^cd^0.18 ± 0.02^B^
Bayo	^a^4.04 ± 0.88^A^	^bcd^1.08 ± 0.76^B^	^bcd^0.15 ± 0.09^A^	^a^0.26 ± 0.005^A^	^abc^0.72 ± 0.12^A^	^cd^0.15 ± 0.03^B^
Alubia	^a^4.09 ± 0.44^A^	^d^0.73 ± 0.15^B^	^cd^0.14 ± 0.03^A^	^h^0.01 ± 0.01^B^	^bcde^0.51 ± 0.22^A^	^bcd^0.27 ± 0.05^A^
**Variety**	**Ca**		**Mg**		**S**	
	**Cotyledon**	**Seed Coat**	**Cotyledon**	**Seed Coat**	**Cotyledon**	**Seed Coat**
Negro 8025	^abc^0.09 ± 0.002^B^	^de^0.96 ± 0.04^A^	^abcd^0.09 ± 0.02^B^	^a^0.35 ± 0.02^A^	^a^0.17 ± 0.000^A^	^cde^0.050 ± 0.01^B^
Flor de Mayo	^cde^0.08 ± 0.005^B^	^gh^0.67 ± 0.13^A^	^abcd^0.09 ± 0.007^B^	^bc^0.27 ± 0.02^A^	^ab^0.15 ± 0.002^A^	^e^0.000 ± 0.000^B^
Negro San Luis	^f^0.06 ± 0.006^B^	^ef^0.87 ± 0.06^A^	^ab^0.10 ± 0.007^B^	^a^0.38 ± 0.002^A^	^b^0.14 ± 0.01^A^	^cd^0.09 ± 0.07^B^
Higuera Azufrado	^cde^0.08 ± 0.004^B^	^i^0.49 ± 0.02^A^	^ab^0.09 ± 0.02^B^	^a^0.34 ± 0.06^A^	^b^0.14 ± 0.006^A^	^a^0.18 ± 0.02^A^
Moyocoa	^cdef^0.08 ± 0.01^B^	^fg^0.75 ± 0.08^A^	^bcd^0.08 ± 0.007^B^	^dc^0.24 ± 0.01^A^	^bc^0.13 ± 0.02^A^	^bcd^0.09 ± 0.03^A^
Negro Criollo	^f^0.06 ± 0.007^B^	^cd^1.08 ± 0.02^A^	^ab^0.10 ± 0.008^B^	^a^0.36 ± 0.01^A^	^b^0.14 ± 0.009^A^	^de^0.04 ± 0.006^B^
Ojo de Cabra	^a^0.11 ± 0.01^B^	^bc^1.18 ± 0.04^A^	^a^0.11 ± 0.02^B^	^bc^0.29 ± 0.02^A^	^de^0.10 ± 0.02^A^	^ab^0.16 ± 0.06^A^
Cuba-V7	^bcd^0.09 ± 0.004^B^	^de^1.32 ± 0.15^A^	^cd^0.07 ± 0.009^B^	^d^0.19 ± 0.03^A^	^e^0.08 ± 0.008^A^	^cd^0.07 ± 0.03^A^
Pinto Saltillo	^ef^0.07 ± 0.003^B^	^cde^0.99 ± 0.13^A^	^abc^0.09 ± 0.005^B^	^b^0.29 ± 0.01^A^	^cd^0.11 ± 0.01^A^	^cde^0.05 ± 0.03^B^
Negro Orgánico	^ab^0.11 ± 0.006^B^	^a^1.34 ± 0.04^A^	^ab^0.10 ± 0.008^B^	^a^0.34 ± 0.006^A^	^e^0.08 ± 0.00^A^	^cd^0.06 ± 0.04^A^
Negro Jamapa	^cde^0.08 ± 0.001^B^	^ef^0.86 ± 0.07^A^	^d^0.06 ± 0.005^B^	^bc^0.25 ± 0.04^A^	^d^0.10 ± 0.005^A^	^bc^0.10 ± 0.05^A^
Bayo	^def^0.07 ± 0.008^B^	^hi^0.59 ± 0.01^A^	^bcd^0.08 ± 0.004^B^	^bc^0.26 ± 0.02^A^	^e^0.09 ± 0.02^A^	^cde^0.05 ± 0.01^A^
Alubia	^abc^0.09 ± 0.03^B^	^a^1.36 ± 0.16^A^	^a^0.11 ± 0.008^B^	^a^0.38 ± 0.014^A^	^e^0.08 ± 0.01^A^	^cde^0.04 ± 0.01^B^

The means shown in different capital letters per line indicate a significant statistical difference (*p* ≤ 0.05) between the seed coat and cotyledon within each variety. The means shown in lowercase letters per column indicate a significant statistical difference (*p* ≤ 0.05) between bean varieties within each part of the seed. Mean ± Standard Deviation.

**Table 7:** Pearson correlation coefficients of variables evaluated in common bean varieties.

Variable	L*	a*	b*	°h	Chroma	CA	P	Ni	Fe	Zn	Cu	Mn	Ca	K	Mg	N	C	H	S	Protein	TP
L*	1																				
a*	0.38 *	1																			
b*	0.74 **	0.49 **	1																		
°h	−0.88 **	−0.72 **	−0.77 **	1																	
Chroma	0.20	−0.04	0.02	−0.09	1																
AC	−0.14	0.15	−0.14	0.02	−0.12	1															
P	0.065	0.22 *	0.32 *	−0.16	0.01	−0.26 *	1														
Ni	0.07	0.16	0.23 *	−0.07	0.09	−0.63 **	0.27 *	1													
Fe	−0.202	−0.44 **	−0.23 *	0.36 *	−0.04	0.25 *	−0.54 **	−0.27 *	1												
Zn	0.34 *	0.24 *	0.63 **	−0.39 *	−0.03	−0.20	0.39 *	0.11	−0.28 *	1											
Cu	0.21	0.07	0.41 *	−0.19	0.08	−0.68 **	0.51 **	0.61 **	−0.37 *	0.52 **	1										
Mn	0.002	−0.11	0.03	0.05	0.04	−0.80 **	0.43 **	0.61 **	−0.26 *	0.14	0.63 **	1									
Ca	−0.06	−0.14	−0.15	0.11	−0.09	0.72 **	−0.58 **	−0.56 **	0.72 **	−0.25 *	−0.67 **	−0.70 **	1								
K	−0.005	−0.04	0.11	0.02	0.04	−0.43 **	0.26 *	0.38 **	−0.20	0.05	0.38 *	0.62 **	−0.4 *	1							
Mg	0.01	−0.08	−0.05	0.02	−0.07	0.78 **	−0.52 **	−0.63 **	0.48 **	−0.26 *	−0.76 **	−0.81 **	0.85 **	−0.40 *	1						
N	−0.10	−0.08	−0.10	0.11	−0.01	0.10	−0.04	−0.06	0.08	−0.05	−0.12	−0.13	0.06	−0.08	0.05	1					
C	0.014	−0.13	0.06	0.01	−0.10	−0.16	0.12	0.07	0.03	0.18	0.06	0.19	−0.21	0.02	−0.13	0.008	1				
H	0.06	−0.08	0.10	−0.03	0.05	−0.50 **	0.29	0.33 *	−0.31 *	0.30 *	0.40	0.50 **	−0.56 **	0.23 *	−0.54 **	−0.02	0.83 **	1			
S	−0.07	−0.18	0.02	0.07	−0.03	−0.21	0.15	0.08	−0.03	0.09	0.12	0.22 *	−0.22 *	0.07	−0.17	−0.06	0.45 **	0.46 **	1		
Protein	0.05	−0.005	0.10	−0.07	0.05	−0.76 **	0.45 **	0.55 **	−0.51 **	0.26 *	0.65 **	0.74 **	−0.84 **	0.37 *	−0.82 **	−0.11	0.50 **	0.79 **	0.42 **	1	
TP	−0.01	0.24 *	−0.10	−0.12	−0.04	0.88 **	−0.28 *	−0.52 **	0.15	−0.15	−0.57 **	−0.69 **	0.66 **	−0.32 *	0.68 **	−0.68 **	−0.13	−0.47 **	−0.25 *	−0.68 **	1

AC = antioxidant capacity. TP = total phenols. Hue angle = °h. * Significant correlation (*p* < 0.05). ** Highly significant correlation (*p* < 0.05).
